# Spinal dural arteriovenous fistula: a comprehensive review of the history, classification systems, management, and prognosis

**DOI:** 10.1186/s41016-023-00355-y

**Published:** 2024-01-09

**Authors:** Ali Alkhaibary, Ahoud Alharbi, Nada Alnefaie, Hajar Alammar, Alshaymaa M. Arishy, Noor Alghanim, Yazeed M. Aldhfyan, Arwa Albaiahy, Yahya H. Khormi, Wael Alshaya, Saad AlQahatani, Ahmed Aloraidi, Ahmed Alkhani, Sami Khairy

**Affiliations:** 1 College of Medicine, King Saud Bin Abdulaziz University for Health Sciences, Riyadh, Saudi Arabia; 2 King Abdullah International Medical Research Center, Riyadh, Saudi Arabia; 3 Division of Neurosurgery, Department of Surgery, King Abdulaziz Medical City, Ministry of National Guard - Health Affairs, Riyadh, Saudi Arabia; 4 Department of Neurosurgery, National Neurosciences Institute, King Fahad Medical City, Riyadh, Saudi Arabia; 5 College of Medicine, Jazan University, Jazan, Saudi Arabia; 6 Department of Neurosurgery, King Faisal Specialist Hospital and Research Center, Riyadh, Saudi Arabia; 7 Department of Surgery, Faculty of Medicine, Jazan University, Jazan, Saudi Arabia

**Keywords:** Connection, Endovascular, Myelopathy, Vascular

## Abstract

Spinal dural arteriovenous fistulas account for the majority of spinal vascular malformations. They are typically located in the thoracolumbar region and are diagnosed in the middle-aged and elderly populations. Although spinal dural arteriovenous fistulas have been postulated to be acquired, their exact development remains uncertain. Typically, the arteriovenous shunt is situated close to the spinal nerve root, inside the dura mater, where the blood from the radiculomeningeal artery and radicular vein intermix. Throughout history, there have been multiple classification systems of spinal arteriovenous shunts since 1967. Those were mainly based on the evolution of diagnostic studies as well as the treatment of these lesions. Such classification systems have undergone significant changes over the years. Unlike intracranial dural arteriovenous fistula, spinal dural arteriovenous fistula is progressive in nature. The neurological manifestations, due to venous congestion, tend to be insidious as well as non-specific. These include sensory deficits, such as paresthesia, bilateral and/or unilateral radicular pain affecting the lower limbs, and gait disturbances. Spinal dural arteriovenous fistulas can be suspected on magnetic resonance imaging/magnetic resonance angiography and confirmed by digital subtraction angiography (DSA). The management includes surgery, endovascular therapy, and in selected cases, radiotherapy. The treatment goal of spinal dural arteriovenous fistula is to halt the progression of the disease. The prognosis depends on both the duration of symptoms as well as the clinical condition prior to therapy. The present article comprehensively reviews the pathophysiology, changes in classification systems, natural history, clinical manifestations, radiological features, management, and prognosis.

## Background

Spinal dural arteriovenous fistulas account for the majority of spinal vascular malformations [1]. They are typically located in the thoracolumbar region and are diagnosed in the middle-aged and elderly population [1]. Although they are considered the most commonly identified spinal vascular malformation, their occurrence is rare and may remain undiagnosed [1]. Underreporting of such clinical entities can render patients with spinal dural arteriovenous fistulas susceptible to paraplegia or tetraplegia [1].

### Etiology and pathophysiology

Although spinal dural arteriovenous fistulas have been postulated to be acquired, their exact development remains uncertain [1, 2], multiple factors have been suggested to play a role in their development [3]. Re-opening of the thrombosed/occluded spinal radicular veins may contribute to spinal dural arteriovenous fistula [3, 4] Understanding the pathophysiology of spinal dural arteriovenous fistula facilitate identifying risk factors and etiologies of spinal dural arteriovenous fistula.

The onset of spinal dural arteriovenous fistulas in the middle-aged population suggests an acquired disease process [2]. The arteriovenous shunt is commonly situated close to the spinal nerve root, inside the dura mater, where the blood from the radiculomeningeal artery and radicular vein intermix [1, 2]. The elevation in pressure causes “arterialization” of the venous system, i.e., thickening of the intramedullary veins [2]. As the radicular and intramedullary veins share a common venous outflow, the shunt consequently becomes congested, causing venous hypertension in the spinal cord [2, 5]. In turn, it leads to decreased tissue perfusion and venous infarction [2, 5].

## Classification systems

Spetzler et al. proposed a modified classification system for spinal arteriovenous lesions [6]. The classification is based on the pathophysiological and anatomical factors [6]. Spinal arteriovenous fistulas were divided into extradural and intradural lesions. Extradural fistulas have a well-described pathophysiology and clinical presentation. A high-flow fistula is formed by a connection between the extradural artery and vein. This causes engorgement of the epidural venous system and compression on the spinal cord, leading to myelopathy. Conversely, the origin, pathophysiology, and management of intradural fistulas remain unclear. Intradural fistulas are divided to dorsal and ventral lesions. Intradural dorsal fistulas are the most common spinal arteriovenous fistulas and are predominantly present in the thoracic region. Intradural ventral fistulas originate from the anterior spinal artery which directly connects to a large venous network [7, 8]. The blood flow is rapid and may lead to venous hypertension and aneurysms.

### Historical changes in classification

**Fig. 1:**
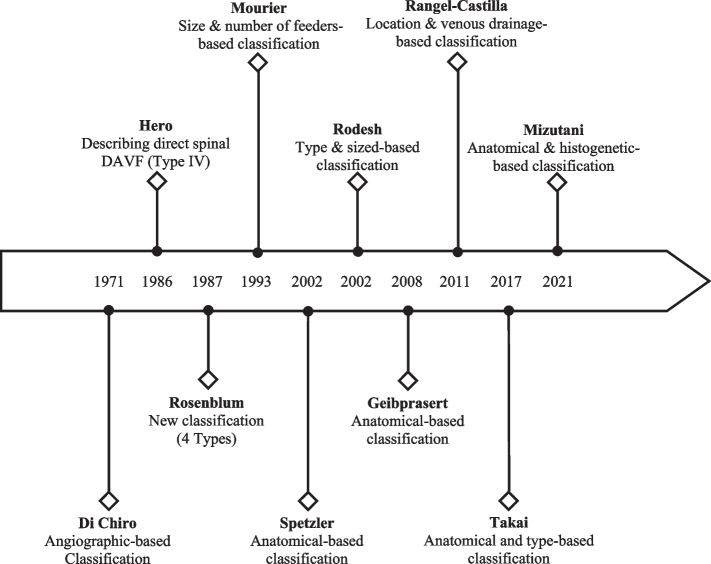
Historical evolution of classification systems of spinal dural arteriovenous fistula

**Fig. 2:**
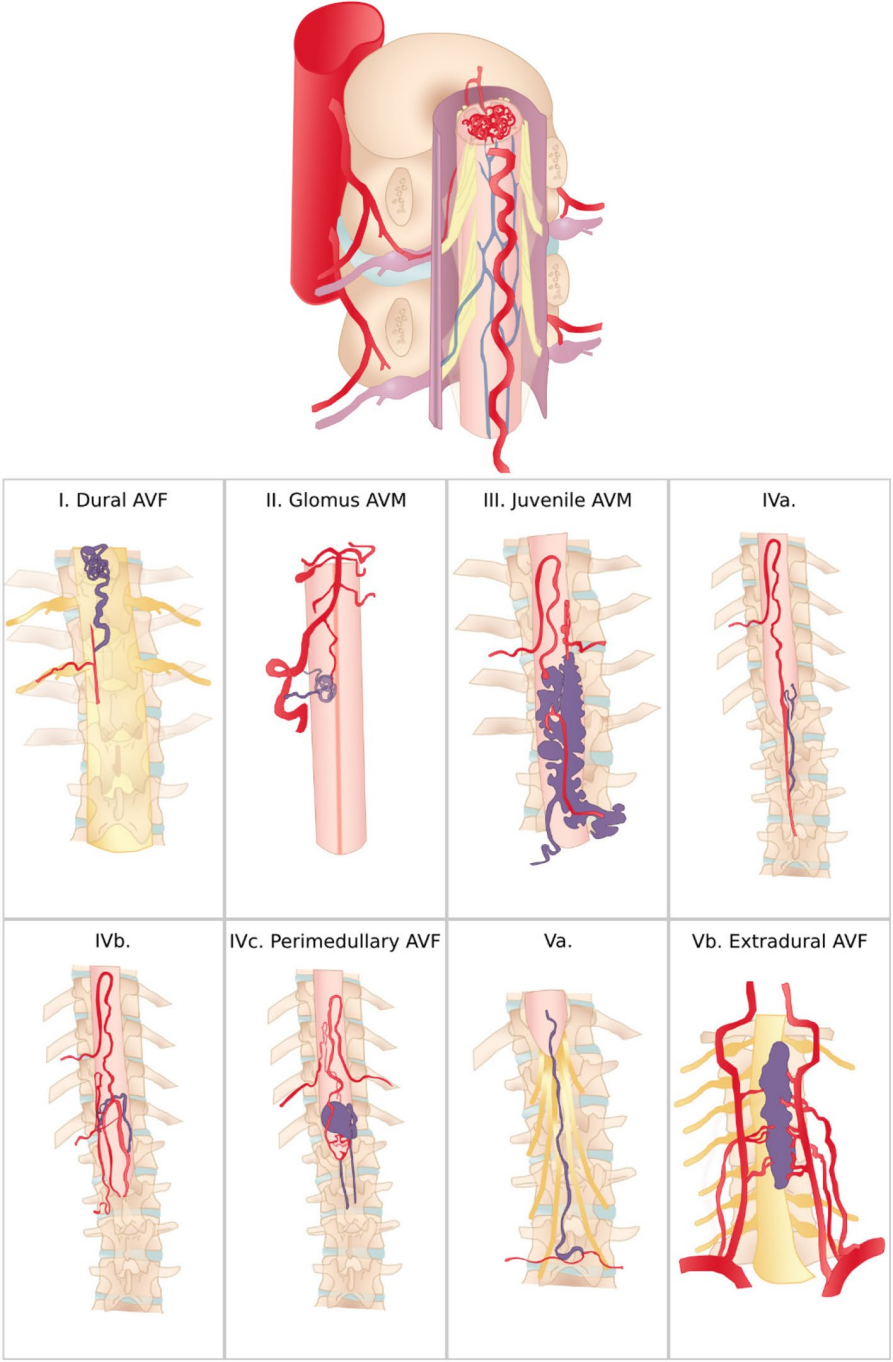
Illustration of the classification systems of spinal arteriovenous fistula

There have been multiple major classification systems of spinal arteriovenous shunts between 1967 and 2021 (Figs. 1 and 2) [9]. Those were mainly based on the evolution of diagnostic studies as well as the treatment of these lesions [9]. The first classification system was introduced by Di Chiro G and his colleagues in 1971 [9]. The classification was based on angiographic findings: single-coiled vessel type I, glomus type II, and juvenile type III [9].

In 1986, Hero RC and his colleagues reported a new type of spinal arteriovenous shunt which did not fit in the first classification system [9, 10]. They reported a case of a patient who had a direct connection between the anterior spinal artery and vein which was located ventrally [9, 10]. That shunt was described as a direct spinal arteriovenous fistula type IV [9, 10].

Rosenblum B reported another classification system in 1987 which classified spinal arteriovenous fistula shunts into four types: type I as dural arteriovenous fistula; type II as intramedullary glomus arteriovenous malformation; type II as intramedullary juvenile arteriovenous malformation; and type IV as direct arteriovenous fistula [9, 11].

In 1993, Mourier KL and Merland JJ reported a series of intradural direct arteriovenous fistulas in which they were described as perimedullary arteriovenous fistulas [9, 12]. It was classified into three subtypes based on the number of feeding arteries and the size of the arteriovenous fistula [9, 12]. Type I with a single feeding artery and a small arteriovenous fistula, Type II with multiple feeding arteries and a medium arteriovenous fistula, and Type III with multiple feeding arteries and a giant arteriovenous fistula [9, 12].

Another classification system was reported by Spetzler RF in 2002 who classified arteriovenous shunts based on the anatomical locations [6, 9]. Dural arteriovenous fistulas were defined as dorsal arteriovenous fistulas since they commonly develop at the dorsolateral aspect of the dura [6, 9]. On the other hand, perimedullary arteriovenous fistulas commonly develop at the ventral portion, they were defined as ventral arteriovenous fistulas [6, 9]. Intramedullary glomus arteriovenous malformations were defined as compact arteriovenous malformations, and intramedullary juvenile arteriovenous malformations as extradural-intradural arteriovenous malformations [6, 9].

Additionally, three new types were defined: extradural arteriovenous fistulas, diffuse type arteriovenous malformations, and conus arteriovenous malformations [6, 9]. In the same year, Rodesh G and Lasjaunias P reported a new classification system based on the type and size of spinal arteriovenous shunts [9, 13]. It was divided into arteriovenous malformations and arteriovenous fistulas, with the latter being subdivided into macro and micro arteriovenous fistulas [9, 13]. Both classification systems were not universally accepted because both were biased toward either microsurgery or endovascular treatment [9].

In 2008, Geibprasert classified the cranio-spinal arteriovenous shunts into three types; ventral, dorsal, and lateral epidural groups [9, 14]. In 2011, Rangel-Castilla reported an endovascular case series of extradural arteriovenous fistula which were subdivided into three types: type A is an extradural arteriovenous fistula with intradural venous drainage, type B1 is without intradural venous drainage but with neurological deficits, type B2 is without intradural venous drainage and without neurological deficits [9, 15].

In 2017, Takai. K proposed a practical classification system based on the classic second classification which has been the most widely used in the literature [9]. Spinal dural arteriovenous fistulas were classified into five categories: type I, the dural AVF, type II, the intradural intramedullary glomus AVM, type III, and the intradural intramedullary juvenile AVM, type IV, the perimedullary AVF, and type V, the extradural AVF [9]. According to the size of the AV shunts, the drainage system, and the feeding artery, perimedullary AVFs, and extradural AVFs are divided into subtypes in this classification system [9]. The advantage of this sub-classification is that different treatment approaches may be suggested depending on the subtypes [9]. Type IVa perimedullary AVFs are more amenable to microsurgery and type IVc perimedullary AVFs to endovascular embolization. Type Vb extradural AVFs are more responsive to endovascular embolization than type Va extradural AVFs, which can be treated with microsurgery and endovascular embolization [9]. Because it is based on the microvascular anatomy and hemodynamics of AV shunts, the proposed classification system is appropriate for developing treatment strategies using microsurgical and/or endovascular treatments [9].

In 2021, Mizutani et al. proposed a novel classification system that is primarily based on the anatomical disposition, angioarchitecture, and histogenetic location of the spinal dural arteriovenous fistulas [16]. A total of five phenotypes were proposed in the classification system [16]. These included: Macro arteriovenous fistulas, micro arteriovenous fistulas, pial niduses, intramedullary lesions, and sulcal lesions [16].

## Natural history

Unlike intracranial dural arteriovenous fistula, spinal dural arteriovenous fistula is progressive in nature [17]. Patients are less likely to present with hemorrhage, if untreated [18]. Symptomatic patients are expected to experience progressive deterioration and cord atrophy in advanced stages of the disease [18]. The symptoms, however, are non-specific which might lead to delayed presentation and diagnosis [1].

If left untreated, spinal dural arteriovenous fistula can lead to severe morbidities like progressive myelopathy as well as bladder and bowel dysfunction [19]. It was estimated that 50% of untreated patients would become severely disabled within 3 years of the onset of symptoms [20]. A combination of sacral segment disturbances, motor, and sensory deficits have been reported in two-thirds of patients with spinal dural arteriovenous fistula at the time of presentation [21]. Additionally, the pre-operative neurological status and the duration of symptoms before presentation are important predictors of the treatment outcomes in symptomatic patients [20].

## Clinical manifestations

The neurological manifestations of venous congestion tend to be insidious as well as non-specific [1] These include sensory deficits, such as paresthesia, bilateral and/or unilateral radicular pain affecting the lower limbs, and gait disturbances [22]. Non-radicular lower back pain has also been frequently described in patients with spinal dural arteriovenous fistulas [1] Late in the course of the disease process, dural arteriovenous fistulas can cause erectile dysfunction and bladder/bowel incontinence [1]. Although dural arteriovenous fistulas present progressively, the interval between the initial neurological deficits and diagnosis can be as acute as one day [23]. Of note, spinal dural arteriovenous fistulas, if located in the cervical-medullary junction with an ascending venous route, may rarely present with subarachnoid hemorrhage [24].

### Radiological features

**Fig. 3:**
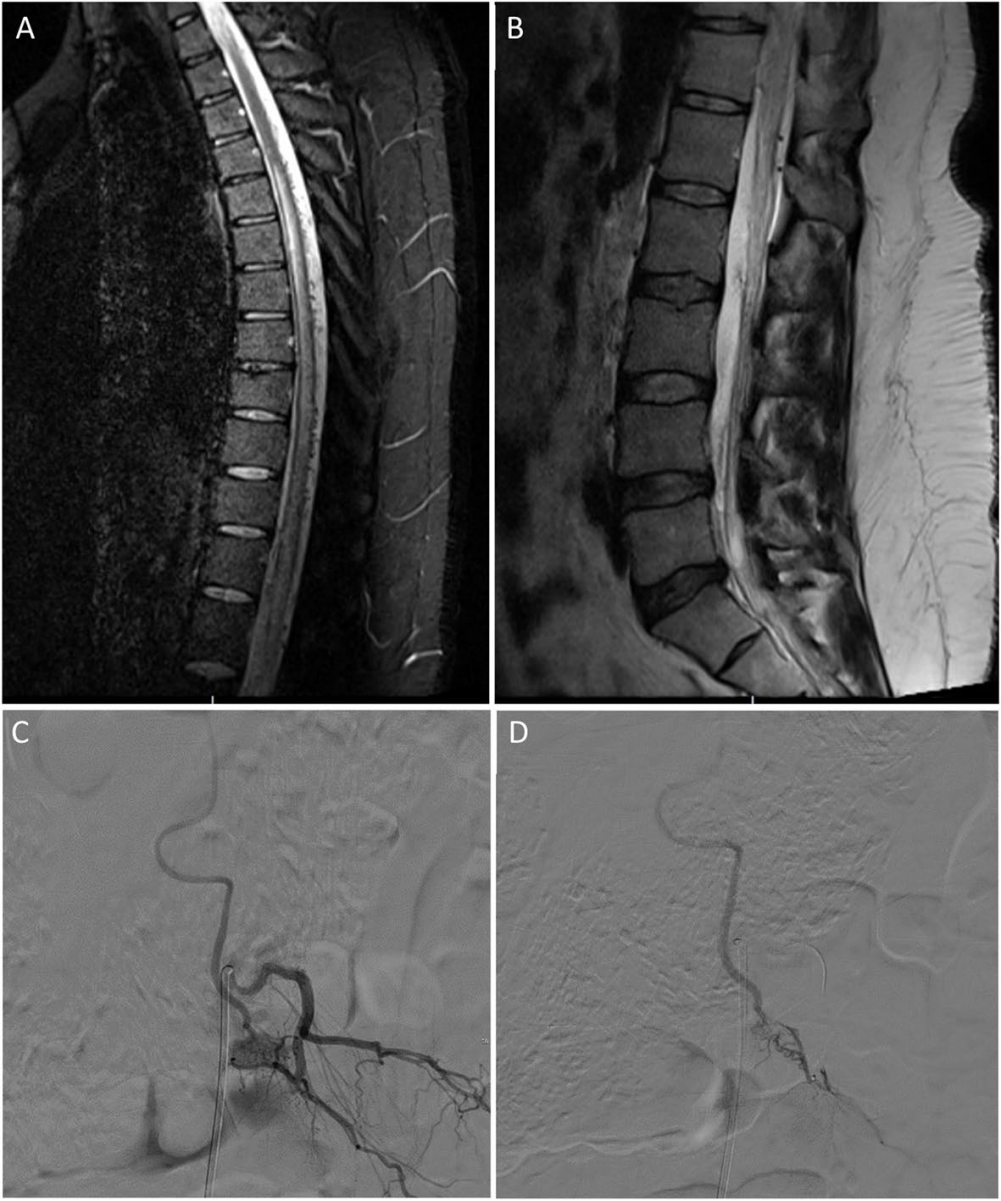
**A**, **B** Sagittal thoracic and lumbar spine MRI demonstrating diffuse long segment spinal cord signal abnormality noted predominantly central in location with mild cord expansion seen starting from T4 vertebral body up to the conus medullaris. The cord shows a central hyperintense signal with peripheral hypointensity and multiple vessels surrounding the cord intradural in location along the lower thoracic spine. **C**, **D** Angiogram images of a left L2 dural AVF (diagnostic run and selective microcatheter run). Embolization was performed using glue

Spinal dural arteriovenous fistulas can be suspected on magnetic resonance imaging/magnetic resonance angiography and confirmed by digital subtraction angiography (Fig. 3) [1, 25].

### Magnetic resonance imaging

On T2-weighted images, the spinal cord displays hyper-intense signals over several segments, coupled with a hypo-intense rim representing the deoxygenated blood within the vessels [26]. T2-weighted MRI is the most sensitive imaging modality to prompt the diagnosis of spinal dural arteriovenous fistulas [26]. On T1-weighted images, the cord may become enlarged and display a hypo-intense signal [26]. Furthermore, diffuse enhancement, likely representing chronic congestion, of the spinal cord can be observed in contrast-enhanced images [1].

### Angiography

On angiography, a normal venous return, post-contrast injection of the anterior spinal artery, can virtually exclude spinal dural arteriovenous fistulas [27]. After contrast injection, stasis of the material can be visualized within the anterior spinal artery. The delay in venous return following contrast administration often indicates venous congestion which necessitates the search for a shunting lesion [1].

## Management

### Surgical management

To the best of our knowledge, the first surgical management of spinal dural arteriovenous fistulas was performed in 1916 by Elsberg [28]. The surgical techniques for treating spinal dural arteriovenous fistulas have evolved over the decades and are mainly centered upon occlusion of the intradural vein receiving blood from the shunt. With the exception of sacral fistulas, this surgical intervention is relatively safe and simple [29]. Microsurgical occlusion of spinal dural arteriovenous fistula yields excellent results [30]. As paraplegic patients may improve following surgical treatment of spinal dural arteriovenous fistulas, attempting aggressive surgical treatment can be reasonable even in patients with a complete loss of the spinal cord function [30].

Definitive interruption of the draining vein and not the arterial feeders is crucial for the success of treatment in order to achieve symptomatic improvement and prevent neurological deterioration caused by recurrence [31, 32]. If not, the fistula has a propensity to re-establish flow by recruiting new arterial feeding vessels, resulting in clinical recurrence [31, 32]. Surgery is still superior and has a higher success rate for fistula obliteration than embolization [32, 33].

Surgical occlusion is considered the mainstay, most definitive, and curative treatment modality for spinal dural arteriovenous fistula, due to its safety, effectiveness, and improvement of neurologic symptoms in most patients. The data also indicate that surgical treatment provides long-term stability after treatment [33–35]. According to the number of previous studies, the highest treatment success rate and low complication rate of surgery may be explained by advances in intraoperative microscopy and neurosurgical techniques over the past decade [34, 36].

Because it is well known that spinal dural arteriovenous fistula can have a high rate of recurrence and that recanalization of the fistula can lead to secondary clinical deterioration, early definitive treatment is required [32]. A recent study found that a shorter duration of symptoms was associated with improved clinical results [32].

Steinmetz and collaborators recommended surgical treatment as the first-line therapy in dealing with spinal dural arteriovenous fistula, because it has a successful occlusion rate of 98%, with 2% morbidity and no mortality [31, 37].

If embolization fails or is not possible due to anatomy-related issues, surgical therapy can be considered as a standby and is often successful in curing patients with low morbidity [32, 38] Surgery should be considered in cases where the selective introduction of the microcatheter is challenging, endovascular treatment is contraindicated due to the presence of an anterior spinal artery originating from the radicular artery feeding the fistula, multiple small feeding arteries are present, embolization failure in the first time or recurrence, endovascular treatment is contraindicated due to extensive atherosclerotic lesions, or recurrence after embolization [31, 37].

In 1984, Symon L and colleagues published a series of 50 surgically-managed patients over 30 years; the aim of their surgery was disconnection of the arterialized vein from the coronal venous plexus and, where possible, obliteration of the arteriovenous malformation [39, 40]. The seven patients who were made worse with surgery all underwent resection of their coronal venous plexus; this technique is no longer recommended in cases of spinal dural arteriovenous fistula because it has the potential to disrupt the venous drainage of the cord [39, 40].

### Endovascular management

In 1968, the first embolization of spinal dural arteriovenous fistula was performed by Doppman et al. [41]. Currently, endovascular treatment is often offered as a first-line therapy of dural arteriovenous fistulas of the spine [42]. Endovascular treatment carries a low-morbidity rate and high chances of cure [42]. If the initial endovascular treatment fails, surgery or repeat angiography/embolization might be offered [42].

Embolization is less invasive than surgery and can be performed in the same setting as spinal angiography [43]. The success rate of endovascular management ranges between 70 and 89.5%. [44]. In one series of 61 patients treated with N-Butyl-2-cyanoacrylate that was published by M. Kirsch and his colleagues, the success rate was 77% [44]. The literature also shows that the endovascular treatment group still has a significant failure rate of 23%, although this percentage has been steadily falling over the past decade due to developments in interventional neuroradiology experience and techniques [44].

As long as the artery of Adamkiewicz does not arise from the same segmental artery as the radiculomeningeal artery feeding the fistula, embolization is a safe procedure for lumbar lesions [43]. A relative contraindication to embolization is when the same segmental artery supplying the fistula contributes to the posterior spinal artery [43]. Potential candidates for endovascular therapy include patients who do not have a common origin for the arteries supplying the spinal cord and the dural fistula, as well as those who do not have stenosis or occlusion of the concerning segmental artery [44].

Endovascular intervention has developed from an adjunct to a potential alternative to surgery [34]. The number of lesions that can be considered for endovascular intervention has risen because of improved embolic agents [34]. This technique's effectiveness and overall durability are still inferior to surgical occlusion [34].

The use of particle embolization is not recommended due to its high recanalization rates; however, the use of liquid embolization material is essential to prevent recanalization [43, 45]. The penetration of the adhesive material to the proximal vein during angiography demonstrates the success rate of endovascular treatment [44]. Discontinuous and uneven glue distribution between the arterial and venous segments tends to cause the fistula to recur [32].

Since the treatment is performed under general anesthesia, better imaging is feasible, especially in the thoracic spine where respiration can produce several artifacts [43]. Diagnostic angiography is performed prior to embolization in order to fully delineate the anatomy of the vasculature surrounding the lesion and to select the best feeder to approach and embolize the fistula [44, 45]. After embolization, these vessels should be checked again to rule out the presence of any fistula remnants [44, 45].

A transfemoral approach is used for most patients [44]. The appropriate catheter is selected for the guide catheter and is designed to achieve the best stability in the involved segmental artery during the procedure, considering the diameter of the aorta and the angle of origin of the segmental arteries [44]. The tip of the microcatheter is coaxially positioned as close as possible to the fistula [44].

Failure to position the microcatheter in the wedge position and placing the microcatheter too far from the lesion as a result of navigation difficulties are the two main causes of treatment failure [44, 45]. Microsurgical treatment is recommended if the arteriovenous shunt persisted after one or more endovascular procedures [44].

When performed by an experienced team, endovascular management has a very low morbidity rate [45]. Additionally, because endovascular treatment is less invasive than open surgery, the postoperative period is less painful, and the hospital stay is shorter [45].

### Surgical vs. endovascular therapy

Based on the largest and most recently reported multicenter cohort study, which was published in 2020, surgery is superior to endovascular treatment for the complete obliteration of spinal dural arteriovenous fistulas [46]. In another study, the success rate of endovascular therapy was reported to be variable between 25 and 75% as compared to 98% which was observed following surgery [1, 42]. Although surgery is often associated with a complete cure, there has been a shift towards endovascular embolization of spinal dural arteriovenous fistulas which is currently considered to be the first-line treatment [47].

Of note, both endovascular and surgical treatment of spinal dural arteriovenous fistulas have resulted in long-lasting and good clinical outcomes in the majority of cases [31]. A multidisciplinary approach based on close collaboration between endovascular specialists and neurosurgeons is required to determine the best initial treatment method for each patient with this type of lesion in order to provide optimal care, which will in turn ensure better clinical outcomes [31, 37, 48]. In the current literature, controversies still exist regarding the best treatment modality for spinal dural arteriovenous fistulas; some authors favor surgery, whereas others recommend endovascular treatment [37].

### Radiotherapy

Although the literature described a case of a spinal dural arteriovenous fistula which was treated successfully with stereotactic radiosurgery, until this time, it is not considered an established treatment option for such lesions, and reports regarding the use of stereotactic radiosurgery in these cases are very limited [49].

Of note, a high expression state of endothelial progenitor cells (EPCs) was found in the cranial and spinal arteriovenous malformation (AVM) tissue, indicating that radiosurgery is not an impossible therapeutic alternative option [50–52]. The results of an experimental study showing that radiosurgery reduces angiogenic activity in AVM tissue compared to untreated AVM tissue have been published by other authors [50]. In comparison to the non-radiosurgical model, stereotactic radiosurgery for an artificial animal AVM showed a reduction in the size of the lesion [50]. Although the results of these studies do not apply to spinal dural arteriovenous fistula, it is hypothesized that radiosurgery could be used to treat spinal dural arteriovenous fistula [50].

According to some studies, the use of stereotactic radiosurgery for arteriovenous fistulas induces smooth muscle expansion, adventitial fibrosis, and an intimal response of arterial feeders, and eventually resulted in fistula obliteration [50, 53]. However, the mechanisms underlying the treatment's outcome were not clearly demonstrated [50, 53].

The arterialized fistula has to be a part of the stereotactic radiosurgery target. Since spinal dural arteriovenous fistulas are located at the nerve root’s sleeve in the intradural space, the target area should be planned to also include the dura margin [50, 51].

## Prognosis

The treatment goal of spinal dural arteriovenous fistula is to halt the progression of the disease, and the prognosis depends on both the duration of symptoms as well as the clinical condition prior to therapy [42]. After complete occlusion of the fistula, approximately two thirds of the patients would have regression of their motor symptoms and only one third would show improvement in their sensory disturbances [42]. Sphincter disturbances and impotence are rarely reversible, and variable degrees of pain may also persist [42].

A retrospective study was published in 2012 to evaluate the clinical outcome of patients treated for spinal dural arteriovenous fistulas [20]. The study included 65 patients who were treated with either surgery or endovascular therapy in three Neurosurgery Departments between 1989 and 2009 [20]. It has been shown that 80% of patients reported improvement of at least one symptom [20]. Motor symptoms were reported to have the best prognosis followed by sensory disturbance, pain, and sphincter dysfunction [20].

## Conclusion

Spinal arteriovenous fistulas are rare; however, they might be under-reported throughout the literature. In spite of our current understanding of spinal dural arteriovenous fistulas, there are still areas of uncertainty in terms of the pathophysiology, imaging modalities of choice, and optimal management. Future, well-defined, prospective studies can facilitate providing evidence-based answers for topics of controversies.
